# Dynamics of plasmid-mediated niche invasion, immunity to invasion, and pheromone-inducible conjugation in the murine gastrointestinal tract

**DOI:** 10.1038/s41467-022-29028-7

**Published:** 2022-03-16

**Authors:** Helmut Hirt, Kerryl E. Greenwood-Quaintance, Aaron M. T. Barnes, Melissa J. Karau, Lisa M. Till, Elise Palzer, Weihua Guan, Michael S. VanNieuwenhze, Purna C. Kashyap, Robin Patel, Gary M. Dunny

**Affiliations:** 1 Department of Microbiology and Immunology, Medical School, University of Minnesota, Minneapolis, MN USA; 2 Division of Clinical Microbiology, Department of Laboratory Medicine and Pathology, Mayo Clinic, Rochester, MN USA; 3 Division of Gastroenterology and Hepatology, Department of Medicine, Mayo Clinic, Rochester, MN USA; 4 Division of Biostatistics, School of Public Health, University of Minnesota, Minneapolis, MN USA; 5 Department of Chemistry, Indiana University, Bloomington, IN USA; 6 Division of Infectious Diseases, Department of Medicine, Mayo Clinic, Rochester, MN USA

**Keywords:** Microbial ecology, Cellular microbiology

## Abstract

Microbial communities provide protection to their hosts by resisting pathogenic invasion. Microbial residents of a host often exclude subsequent colonizers, but this protection is not well understood. The *Enterococcus faecalis* plasmid pCF10, whose conjugative transfer functions are induced by a peptide pheromone, efficiently transfers in the intestinal tract of mice. Here we show that an invading donor strain established in the gastrointestinal tract of mice harboring resident recipients, resulting in a stable, mixed population comprised of approximately 10% donors and 90% recipients. We also show that the plasmid-encoded surface protein PrgB (Aggregation Substance), enhanced donor invasion of resident recipients, and resistance of resident donors to invasion by recipients. Imaging of the gastrointestinal mucosa of mice infected with differentially labeled recipients and donors revealed pheromone induction within microcolonies harboring both strains in close proximity, suggesting that adherent microcolonies on the mucosal surface of the intestine comprise an important niche for cell-cell signaling and plasmid transfer.

## Abstract (web summary)

Microbial communities provide protection to their hosts by excluding colonizing pathogens. Here the authors study plasmid transfer and plasmid-mediated effects on host colonization and persistence of *Enterococcus faecalis* in the intestinal tract of mice.

## Introduction

Mobile genetic elements, including conjugative plasmids, are generally viewed as a burden to their host bacteria under non-selective conditions^1^ with the cost/benefit ratio associated with plasmid carriage being a key determinant of success over evolutionary time scales. Plasmid-encoded antibiotic resistance determinants and other “cargo” genes coding for special properties are often associated with transposable elements that may have invaded the plasmid recently in the evolutionary history of the core plasmid genome; knowledge of the contributions of these core genomes to host fitness is limited. In contrast to most other plasmid systems whose transfer has been analyzed in vivo, the transfer functions of the enterococcal pCF10 plasmid are activated in donor cells by peptide signals from recipients^2^.

The term invasion can have several ecological connotations: here we follow the definitions of Richardson^3^ and Daehler^4^ where a successful invasive population can self-sustain and spread within an environment. Here, we characterized two types of invasion, (1) the ability of *Enterococcus faecalis* strains with or without the conjugative plasmid pCF10 to occupy an environment already colonized by a closely related strain, and (2) the transfer of pCF10 to a previously plasmid-free recipient population. This work shows that pCF10 carriage enhances both the ability of a donor strain to invade a resident recipient community, and the ability of donor residents to exclude invading recipients. Imaging analysis suggests that microcolonies on the GI mucosal surface represent an important niche for colonization, pheromone induction of donor cells by adjacent recipients, and plasmid transfer.

## Results

### The pCF10 plasmid transfers in vivo, and promotes both niche invasion and resistance to invasion

**Fig. 1:**
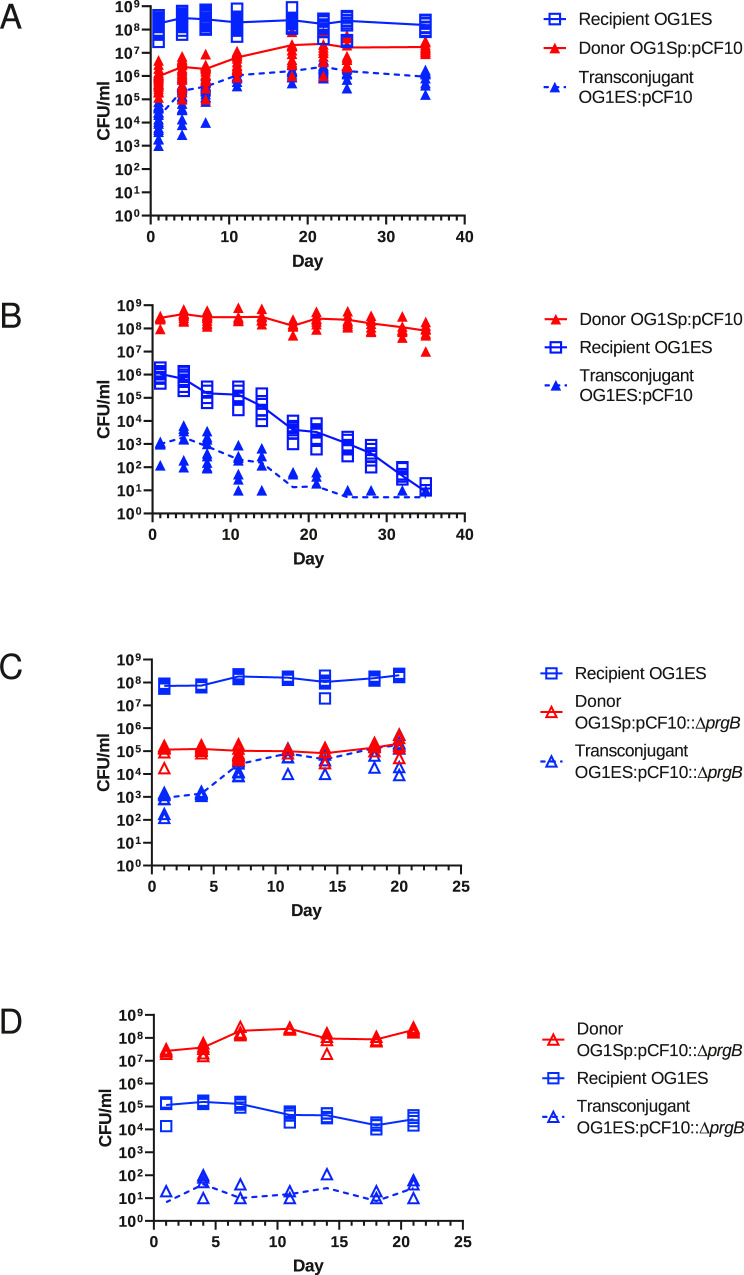
Colonization, niche invasion, and plasmid transfer in mice mono-associated with *E. faecalis* donor/recipient communities. As described in Methods, germ-free mice were initially gavaged with either wild-type recipients (OG1ES) (**A**, **C**) or donors, either OG1Sp:pCF10 (**B**) or OG1Sp:pCF10*ΔprgB* (**D**) to establish a GI resident population. Three days later, the mice were gavaged with the opposite cell type, which comprised the invading strain. The populations of recipients, donors, and transconjugants in feces were enumerated by plating on selective media at various time points from 1–35 days after the addition of the invading strain. Each plotted point represents results from a single mouse. A subset of mice from these experiments was also sacrificed at selected time points and bacterial associated with different sections of the intestine were enumerated, as shown in Supplementary Fig. 2. OG1ES is marked in blue, OG1Sp in red. The presence of plasmid pCF10 is indicated by a triangle (∆), the plasmid pCF10*ΔprgB* is represented by a diamond shape (◊). Statistical analysis for the data presented in Figs. 1 and 2 were performed using Student’s *t* test with Welch’s correction using GraphPad (v. 9) and R (v. 3.53) software. The number of input animals for each experiment are as follows: **A**- 26 (7 cages); **B**- 14 (4 cages); **C**- 8 (2 cages); **D**- 8 (2 cages). Detailed enumeration data are listed in the Source Data File.

We have previously reported that when wild-type pCF10-containing donor cells were introduced into the gastrointestinal (GI) tract of mice harboring a resident *E. faecalis* recipient strain, the plasmid-containing (donor) population increased from <1% to ~10% of the total population within 7 days^5^ (note that the *total* donor population includes invading donors plus transconjugants). These results were surprising given previous studies of other bacterial species where invading donor strains without positive selection were “washed out” of animals harboring resident recipients, even though the plasmid itself was transferred to the resident population^6–8^. To determine whether invading OG1Sp:pCF10 donors could eventually displace plasmid-free OG1ES resident recipients, or if pCF10 would spread through the whole recipient population over a longer time frame, we followed mice for 35 days after the introduction of OG1Sp:pCF10 into mice with resident OG1ES recipients. One day after the addition of donors, ~10^4^ transconjugants (OG1ES:pCF10), 10^6^ donors, and 10^8^ recipients/g feces were recovered (Fig. 1A). We observed that over the entire 35-day experimental course, the recipient population remained between 1–5 × 10^8^, while donor and transconjugant populations increased gradually over the first 21 days, with their numbers not significantly changing after that time point (*p* = 0.155). The stable community of the three cell types at the end of the experiment consisted of about 90% unmated OG1ES recipients, 10% OG1Sp:pCF10 donors, and 0.05% OG1ES:pCF10 transconjugants (Fig. 1A). We found that population dynamics observed in the first week replicated our previous results^5^, and the longer time frame revealed the limit of the extent of invasion of both the pCF10-carrying donor strain and pCF10 itself into the resident recipient community. Both invasion events reached a steady-state by the third week, resulting in a stable mixed enterococcal community of donors, recipients, and transconjugants. The plasmid-free resident recipients remained the dominant genotype; in fact, the initially established resident strain remained as the majority subpopulation at the endpoint of all experiments reported here.

Previous in vitro studies showed strong negative regulation of pCF10 transfer functions in environments with high donor densities^9^. This observation raised the question of whether OG1Sp:pCF10 would transfer pCF10 to invading OG1ES recipients in a niche dominated by a resident donor population. In this situation, we observed strikingly different population dynamics. Population levels of the recipient invaders on day 1 (Fig. 1B) were similar to those observed for donors invading an established recipient population (Fig. 1A), and plasmid transfer from resident donors to recipients was readily detected at this time point, albeit at a lower frequency/donor as discussed below. Populations of both invading recipients and newly formed transconjugants (generated from the invaders) decreased thereafter. Notably, OG1ES:pCF10 transconjugants were undetectable in all mice in this experiment after day 24 and unmated recipients were eliminated in three of eight mice by the end of the experiment on day 35 (Supplementary Fig. 1).

Donor, recipient, and transconjugant populations (mean values for all mice in each group) from the experiments shown in Fig. 1A, B are listed in the top portion of Table 1, together with the ratio of transconjugants per donor, and per recipient, respectively, 1 day after mice with an established resident strain were gavaged with an invading strain. We propose that results are seen at this time point primarily reflect the rate of plasmid transfer, whereas data from later time points could be driven by fitness differences between strains for GI persistence. When we compare total populations of donors, recipients, and transconjugants to transfer frequencies, several conclusions can be drawn from the results. In both experiments 1 A and 1B, the total enterococcal populations were between 10^8^ and 10^9^, whereas populations of residents and invaders at the 1-day time point were similar, with the invaders present at 0.1–1% relative to the residents (Fig. 1A, B). Conjugation frequencies measured as transconjugants per donor (i.e., percentage of donor cells that successfully transferred pCF10) were over 10,000-fold higher at Day 1 when donors invaded recipient residents, whereas the relative Day 1 transconjugant per recipient ratio (percentage of recipient cells that were invaded by pCF10) was higher when pCF10 transferred from the resident donor to the invading recipient. Although the total number of transfer events (based on transconjugant populations) was ~10-fold higher when donors invaded established recipients (Experiment 1 A), 10^3^ transconjugants were still generated when resident donors were in the majority (Experiment 1B) indicating that the pheromone-inducible transfer system of pCF10 has evolved to enable a detectable level of transfer even when donors dominate the community in vivo. Finally, we note that results based on enumeration of adherent bacteria from GI sections in both experiments (Supplementary Fig. 2) were consistent in terms of relative numbers of donors, recipients, and transconjugants in each tissue sample with those from feces presented in Fig. 1 and Table 1; as expected the total bacterial populations in the tissue samples increased from the upper small intestine to the colon.

**Table 1:** Numbers of transconjugants (T), transconjugants/donor (T/D), transconjugants/recipient (T/R), and recipient/donor (R/D) ratio on Day 1 after inoculation of the invading strain.

Experiment	Invader	Resident	T	T / D	T / R	R/D
1A	OG1Sp:pCF10	OG1ES	2.02E + 04	5.02E-02	1.44E-04	2.84E + 02
1B	OG1ES	OG1Sp:pCF10	1.01E + 03	3.96E-06	1.14E-03	3.83E-03
		*p* value	<0.0001	<0.0001	<0.0001	<0.0001
2C	OG1ES:pCF10	OG1Sp	3.40E + 04	5.12E-02	1.83E-04	2.68E + 02
2D	OG1Sp	OG1ES:pCF10	7.39E + 03	4.15E-05	1.91E-03	4.55E-02
		*p* value	<0.0001	<0.0001	0.1106	<0.0001
1A–2C	Donor	Recipient	0.0341	0.1147	0.0948	0.1030
1B–2D	Recipient	Donor	0.0078	<0.0001	0.7345	<0.0001

**Fig. 2:**
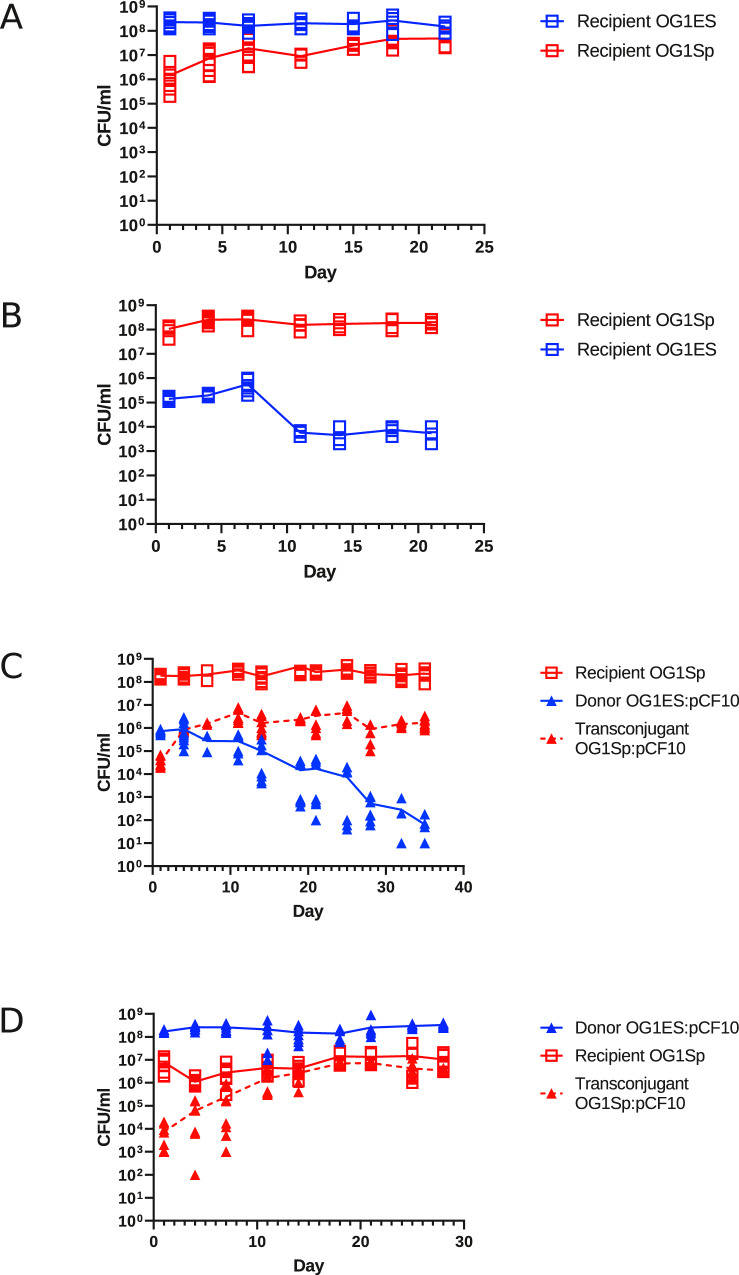
Role of strain background in niche invasion, invasion immunity, and pCF10 transfer. Experiments analogous to those shown in Fig. 1 were carried out (same enumeration methods and data analysis): **A**, **B** show the plasmid-free strain OG1ES and OG1Sp in the resident or invader roles. **C**, **D** are analogous to the experiments shown above in Fig. 1A, B, but with OG1ES as pCF10 donor strain and OG1Sp as plasmid-free recipient. OG1ES is marked in blue, OG1Sp in red; the presence of plasmid pCF10 is indicated by a triangle (∆). Numbers of mice (cages): **A**- 8 (2); **B**- 8(2): **C**- 12(3): **D**- 8(3). Detailed Enumeration data are listed in the Source Data File.

*P* values are given for the pair-wise comparison between the two experiments. Values were tested for normal distribution (Kolmogorov–Smirnov Test) before a Mann–Whitney ranked test. The numbers of animals used were described in the legends to Figs. 1 and 2.

### pCF10 aggregation substance protein PrgB mediates both invasion and resistance to invasion

Our experiments were performed without in vivo selection for pCF10-mediated tetracycline resistance to facilitate the identification of genetic determinants for competitive fitness of pCF10 in the GI tract unrelated to antibiotic resistance. We reasoned that the pheromone-inducible Aggregation Substance protein (PrgB) of pCF10 might impact plasmid transfer and in vivo community invasion, as this surface adhesin has been shown to promote the formation of bacterial aggregates in vitro that increase conjugation and biofilm formation^10,11^, and enhance bacterial attachment to host tissues^12,13^. Invasion by OG1Sp:pCF10*ΔprgB* was reduced when this donor challenged the resident OG1ES community compared to OG1Sp:pCF10 (*p* < 0.001), with the final community after 21 days harboring ~0.1% each of invading donors and transconjugants (Fig. 1C). This outcome shows the strong contribution of PrgB to the success of the invading pCF10-harboring strain and the positive impact of PrgB on the spread of pCF10 in the resident recipient population. The donor population never increased above the 10^5^ cells observed on day 1, (Fig. 1C). When OG1Sp:pCF10*ΔprgB* donors were established as residents (Fig. 1D), populations of residents and invaders were equivalent to those observed in animals harboring a resident recipient population invaded by *ΔprgB* donors (Fig. 1C). In stark contrast to the elimination of the invading recipient by wild-type pCF10 resident donors (Fig. 1B), resident pCF10*ΔprgB* donors (Fig. 1D) were unable to completely exclude the invading OG1ES recipient (*p* < 0.001). These results suggest that both the ability of the donor to invade a resident recipient population and of a resident donor to exclude an invading recipient were directly mediated by PrgB in the OG1Sp strain background. Although the expression of PrgB on donor cells enhances aggregation and plasmid transfer in liquid cultures, PrgB is not required for efficient in vitro transfer on solid surfaces^11^, and the data shown in Fig. 1C, D suggest that in vivo the absence of PrgB limited donor invasion and spread of pCF10*ΔprgB* in the resident recipient population. Notably, the transconjugant populations—and frequencies of transfer—were much lower at all time points for the *ΔprgB* donor.

### Strain genotype and pCF10 carriage both impact invasion in vivo

OG1Sp and OG1ES were both derived from the parental OG1 strain by selection for one-step spontaneous antibiotic resistance; conceivably, these mutations could contribute to the observed population dynamics. Thus, we conducted in vivo competitions involving the plasmid-free strains of each genotype, as well as experiments that involved plasmid-free OG1Sp recipients and OG1ES:pCF10 donors. We identified strain-specific differences in both invasion and exclusion that were separable from those associated with pCF10 carriage.

The plasmid-free OG1Sp strain was able to invade OG1ES residents (Fig. 2A) and form a stable subpopulation (~10%) comparable to OG1Sp:pCF10 donors invading a resident community of OG1ES (Fig. 1A). This suggested higher competitiveness of OG1Sp in vivo, which we also observed in vitro (Supplementary Fig. 3). When the order of inoculation was switched (Fig. 2B), OG1ES invaders were not able to expand, but they did persist at a very low level as a stable subpopulation (~0.1%) within the OG1Sp resident community. The effect of pCF10 on the exclusion of invaders can be assessed by comparing Figs. 1B with 2B, where dynamics of invasion were different depending on the presence of pCF10, and OG1ES invaders were eliminated by OG1Sp:pCF10 residents in the former experiment.

When we carried out experiments equivalent to those depicted in Fig. 1A, B but with the donor chromosomal genotype switched to OG1ES and competing with OG1Sp recipients, two interesting results were obtained. First, when we examined the invasion of resident OG1Sp recipients by OG1ES:pCF10 donors (Fig. 2C), OG1Sp:pCF10 transconjugants were generated rapidly and persisted as an ~1% subpopulation, whereas invading donors were greatly reduced or even eliminated by the end of the experiment. Indeed, population dynamics and establishment of transconjugants in the resident recipient population showed no statistically significant difference between the experiments shown in Figs. 1A and 2C (Table 1), and the stable population contained between 1 and 10% total donors in both experiments. From the data shown in Fig. 2C, we infer that transconjugants formed a stable minority community within unmated recipients, and also outcompeted invading donors owing to differences in chromosomal genotype. When OG1ES:pCF10 donors were established as the initial residents, the invading OG1Sp genotype reached a subpopulation level approaching 10% (Fig. 2D). Intriguingly, when we tested colonies picked from spectinomycin plates (allowing growth of both unmated recipients and transconjugants), 235 of 242 tested exhibited the tetracycline resistance phenotype of pCF10 even though tetracycline resistance was not selected for in vivo or on the primary enumeration plates (pCF10 carriage 94.2 ± 4.5% of OG1Sp population, 95% confidence interval). We conclude that pCF10 carriage confers a competitive advantage in vivo to invading OG1Sp distinct from the fitness effects of the chromosomal genotype.

As shown in Table 1, comparison of the two experiments with the donor in the invader role (1 A: OG1Sp:pCF10; 2 C:OG1ES:pCF10) showed no significant differences between the donor strains in plasmid transfer efficiency or total transconjugants on Day 1, indicating that the chromosomal genotype of the donor did not affect transfer during the 1-day period following invader inoculation. In contrast, a comparison of the experiments involving the two recipient strains as invaders (1B and 2D) showed differences in total transconjugants and transconjugants/donor (Table 1); this likely reflects chromosomally-determined differences in fitness between OG1Sp and OG1ES.

**Fig. 3:**
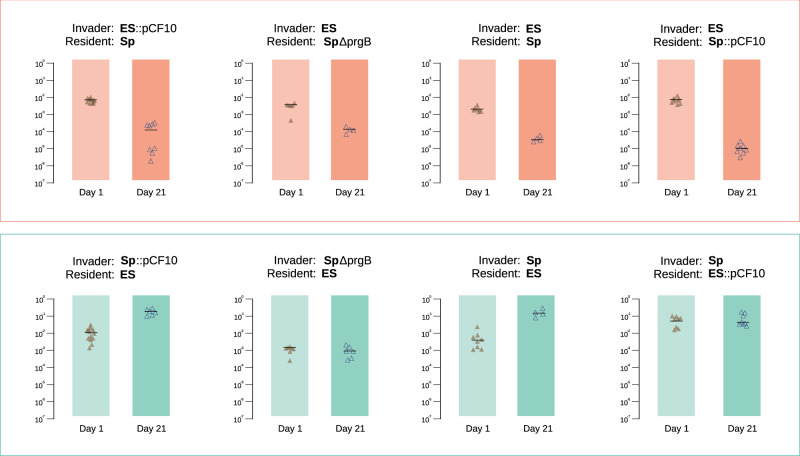
Success as an invader is dependent on strain background, pCF10, and PrgB. The invasion index (II) is defined as the cell number of the invading strain divided by the cell number of the resident strain in feces (the same primary data used to generate Figs. 1 and 2), at either day 1 or Day 21. Statistical analysis was performed by ANOVA, followed by tests for normal distribution and Mann–Whitney ranked test two-tailed for pair-wise comparison. *P* values for the population differences between the time points are as follows (invader/resident): ES:pCF10/Sp- <0.001, ES/SpΔprgB- 0.001, ES/Sp- 0.004, ES/Sp:pCF10- <0.001, Sp:pCF10/ES- <0.0001, SpΔprgB/ES- 0.181, Sp/ES- 0.004, Sp/ES:pCF10- 0.505. Detailed statistical analysis is presented in the Source Data File.

Figure 3 summarizes the strain invasion experiments described above, where each point represents one mouse. The contribution of plasmid pCF10 in the two strain backgrounds OG1Sp and OG1ES to an invasion of a niche occupied by the opposite strain or exclusion of an invading strain are displayed in Fig. 3A as “invasion index” (II). II is defined as the ratio of invaders/residents (on day 1 or day 21) following the introduction of the invading strain. Although Day 1 provides a baseline and gives an indication of the early kinetics of invasion, the Day 21 II reflects the long-term competitive fitness as determined by strain background and pCF10 carriage.

The data depicted in the upper panels of Fig. 3 illustrate the relative fitness defects of all OG1ES strains as invaders, with the index falling between Day 1 and 21 in all cases. In contrast, OG1Sp derivatives were more successful invaders, with indices that either increased or remained constant between Days 1 and 21 (Fig. 3, lower panels). Comparison of Day 21 indices for invading OG1Sp, OG1Sp:pCF10 and OG1Sp:pCF10*ΔprgB* suggests the interesting possibility that, in the absence of a functional *prgB* gene, pCF10 carriage may confer a fitness *cost* to the invading host strain; Day 1 indices also support this notion.

Invasion of pCF10 into the plasmid-free recipient population followed a different pattern and was not significantly dependent on strain background but rather on the role of the recipients as resident or as invader (Table 1). pCF10 invasion into the recipient population at day 1 was highly efficient and reached values observed in vitro. There was no difference between the success of pCF10 invading the resident recipient population if originating in OG1Sp or OG1ES (Table 1). It is notable that the percentage of plasmid-carrying recipients *versus* total recipients increased between Day 1 and 21 in all experiments (Figs. 1A, B and 2C, D). The most striking result was the presence of pCF10 in virtually all remaining OG1Sp invaders at Day 21 (Fig. 2D), a clear indication that selective pressure unrelated to antibiotic selection favors pCF10 carriage in the GI tract.

### Pheromone signaling in vivo

The *prgB* gene is within the long *prgQ* operon of pCF10 and contains over 25 pheromone-inducible genes involved in conjugative plasmid transfer. In vitro, the expression of these genes is tightly controlled by several complex and interdependent mechanisms^14^. Ultimately, the induction state of a donor cell is determined by the ratio of two extracellular signaling peptides, ***C*** (cCF10, encoded by the chromosomal *ccfA* gene), and ***I*** (iCF10, encoded by the pCF10 *prgQ* gene). Both peptides are secreted by donor cells and re-imported into these cells, where they compete for binding to the master transcription regulator PrgX. PrgX-***C*** complexes result in induction of the *prgQ* operon while PrgX-***I*** complexes repress the operon. This control system has evolved under selective conditions that require delicately balancing costs of excessive expression—including growth defects and cell lysis^15^—with the benefits of controlled expression, which still allows the spread of the plasmid to new hosts, and increased competitive fitness of the bacterial host cell, as described above.

The results of previous studies of colonization of gnotobiotic mice by enterococci and of the role of peptide pheromones in pCF10 transfer in the GI tract, suggesting that the bacteria are localized in biofilm-like microcolonies throughout the epithelial surface of the GI tract^16^. Although previous work suggested significant induction of donors in the absence of recipients could occur in an environment where serum is present^17^, it seems unlikely that this could occur in the intestine. Pheromone induction of plasmid transfer in the intestine appears to be limited to donors that are in close physical proximity to recipients since the presence of wild-type ***C***-secreting recipients in the same animals containing mutants unable to produce ***C*** did not restore the conjugation defect of the latter^5^.

**Fig. 4:**
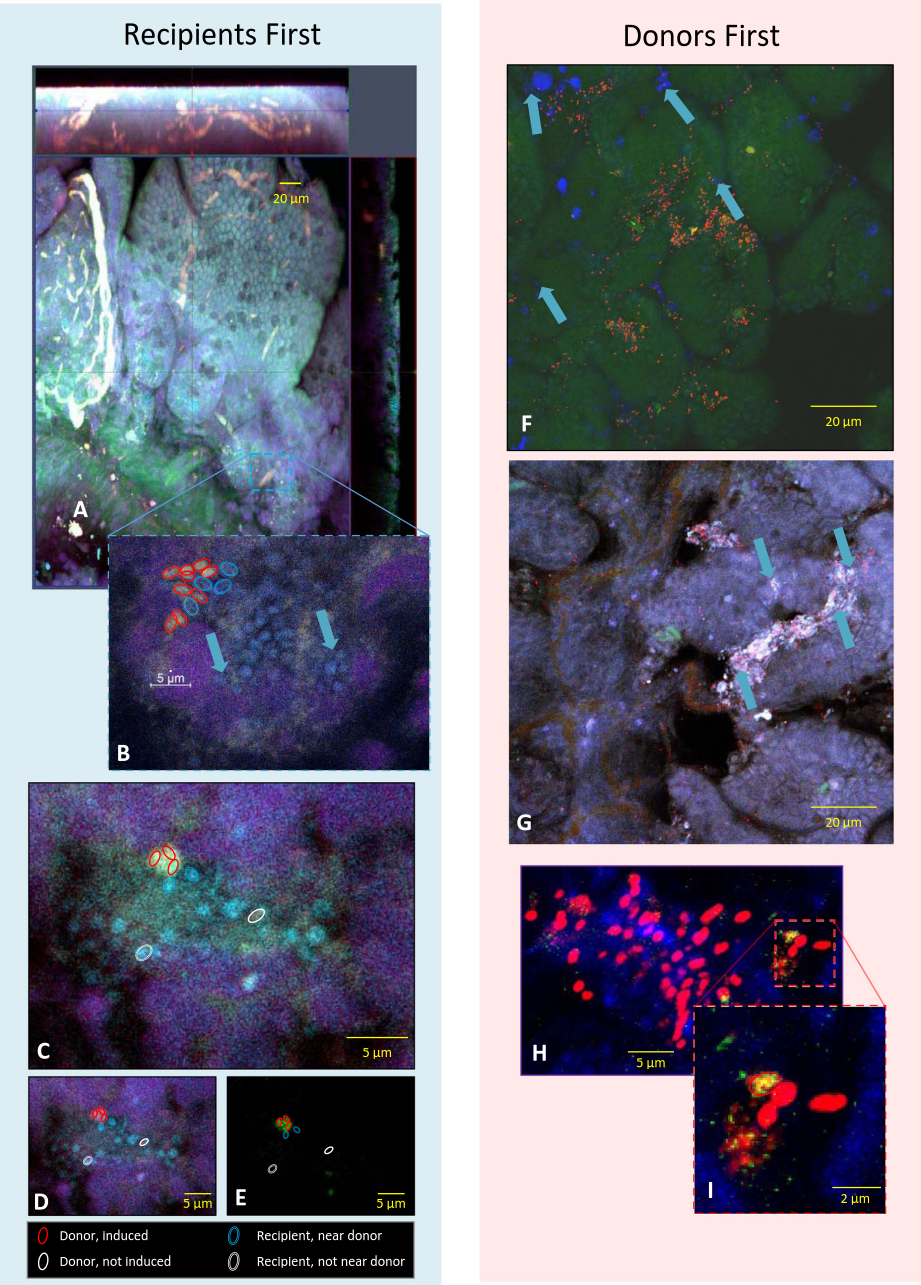
IFM imaging of Recipient (R) and Donor (D) interactions in the murine GI tract. The cell wall of the recipient strain (OG1ES) was pre-labeled with the fluorescent d-amino acid HADA (blue). Donors (OG1Sp) constitutively express the tdTomato fluorescent protein (orange-red) and, when driven by the presence of nearby recipients, inductively expressed GFP (green); for convenience, the color scheme for identification of different cell types is also depicted in the lower-left corner of the figure. Gnotobiotic mice were gavaged with either Recipient first (OG1ES; **A**–**E**) or Donors first (OG1Sp:pCF10; **F**–**I**). After 72 hours, the other strain (D for the R first mice; R for the D first mice) was gavaged and, 6 hours post gavage, the mice were killed. The images shown are representative of nine animals. Donors invade recipients. **A** Villi and microvilli (top) can be seen in this orthogonal projection of a portion of the proximal ileum. **B** At higher magnification, numerous ~1 μm structures labeled in blue (arrows; blue circles) can be seen attached and clustered on the epithelial surface (not all cells are labeled to allow for unrestricted viewing). In addition, some orange-red labeled cells are clustered nearby (red circles). **C** In a matched sample from a different mouse, clusters of blue cells (recipients; HADA) can be seen on the epithelium with a small cluster of yellow cells (red circles). Separating the fluorescent channels into Red+Blue (**D**) and Green only (**E**), reveals that the yellow cells in **C** are composed of cells labeled both red and green, consistent with Donor cells (red) adjacent to Recipient cells (blue) expressing the inducible GFP from the pCF10 plasmid. Notably, there is also at least one Donor cell in this field (**D**: white single outline) that is not expressing GFP (**E**, white single outline), suggesting it has not yet been induced by the presence of neighboring Recipients. Recipients invade donors **F** Numerous donors (red) can be seen in this proximal ileum sample with many fewer recipients (blue; blue arrows) present. **G** A matched sample from another mouse provides a similar field of view: many donors (red) and a few recipients (blue; blue arrows). **H** High magnifications show similar clusters of donors and recipients as seen in **C**–**E**. Although limited in number, **I** appears to show at least one donor (red) that has been induced (red circle) and is beginning to express the inducible GFP from pCF10.

To examine pheromone induction and plasmid transfer at the single-cell level in the GI tract, we visualized the behavior of conjugation-proficient donors and recipients differentially labeled with fluorescent probes in the mouse intestine. Donors carrying pCF10 encoding a constitutively expressed tdTomato fluorescent protein (i.e., always red), whereas induced donors also expressed a pheromone-inducible GFP derivative inserted in the intergenic region between *prgC* and *prgD* (see Methods for details). Fluorescent d-amino acid labeled recipients (blue) were also used in these experiments; we introduced one of the strains into gnotobiotic mice and allowed this strain to establish for 72 h, prior to the addition of the second strain. Mice were sacrificed 6 h after the introduction of the invading strain, a time point that we had previously established as sufficient for detection for plasmid transfer in the murine GI tract in our system^5^. In mice initially colonized by recipients, we observed mucosal surface microcolonies (with nearly all these cells exhibiting blue fluorescence) throughout the GI tract (e.g., Fig. 4A, B) as expected from the previous studies^16^. Invading donor cells were also detected in low numbers in these mice, also in agreement with previous results based on enumeration of strains by plating homogenized tissues on selective media. Figure 4C, E shows an example of a recipient microcolony associated with an invading donor in red; that donor cell was clearly induced as indicated by co-expression of GFP. In mice initially colonized with donors, widespread colonization of mucosal surfaces by both strains was observed, but with donor cells present in much larger numbers, as anticipated (Fig. 4F–I). The red (but not green) fluorescence of most of these cells suggests that they were generally not induced in the GI environment of these mice. Closer inspection revealed some green-labeled (induced) donor cells (Fig. 4G–I). All induced donors identified in these experiments were in close physical proximity to blue-labeled recipient cells, suggesting induction mediated via direct contact (Fig. 4C–I).

**Fig. 5:**
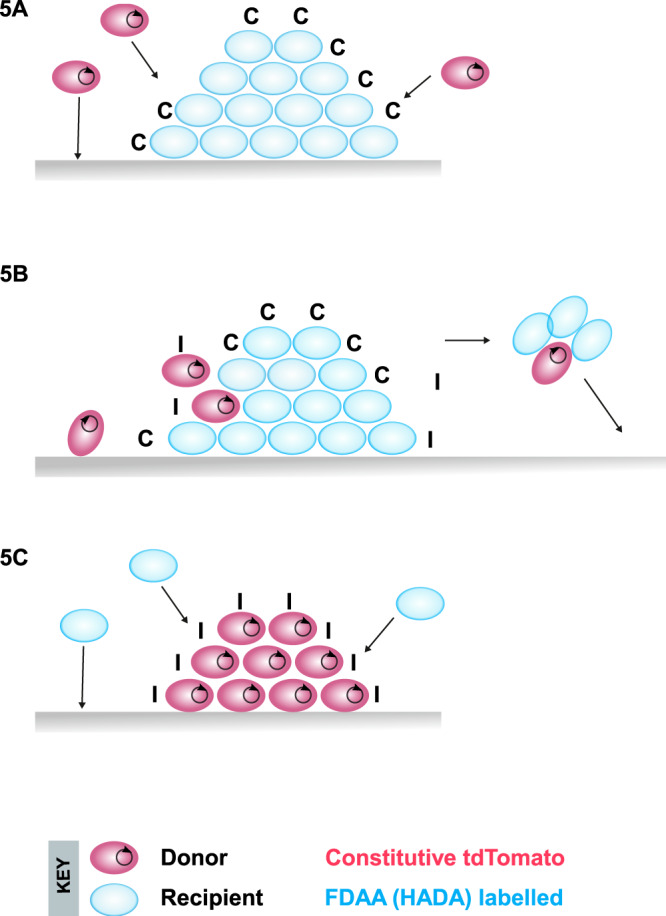
Models for pCF10-mediated conjugation and invasion of resident populations in the mouse intestine. **A** Donors invade recipients Donor cells (blue) in the lumen can attach to recipient microcolonies (red), or directly to the mucosal surface. Because of the high pheromone ***C*** concentration in the colony, donors that attach are induced to transfer plasmids (black circles) to adjacent recipients. The spread of the plasmid through the microcolony is limited by the gradually increasing levels of ***I*** resulting from plasmid dissemination and from the pheromone-induced expression of the *prgQ* gene encoding the ***I*** peptide; this generates a colony comprised of plasmid-containing and plasmid-free cells. **B** Cells and small aggregates from the colony likely re-enter the planktonic phase by direct exit or when the colony is disrupted by turnover of the mucosal surface, and re-attach to generate new microcolonies. **C** Recipients invade donors when recipients invade a niche containing resident donors, the high pre-existing concentration of ***I*** coupled with the low population density of the ***C***-producing invaders minimizes the expression of transfer functions. The rare transconjugants generated probably result from contact-dependent pheromone induction of donors adjacent to the attached recipient. Unless the invading recipients have a plasmid-independent fitness advantage, the transconjugants have no advantage over the resident donors, and they are eventually eliminated, along with the unmated recipients are eliminated. As in **B**. cells and small aggregates from the colonies may re-enter the lumen and then re-attach, but this does not prevent the eventual elimination of the invaders.

The results obtained in the experiments shown in Fig. 1A, B and their visualization in Fig. 4 are consistent with a model of invading donors attaching to recipient microcolonies and where these adherent residents secrete substantial amounts of ***C***—promoting efficient plasmid transfer (Fig. 5). The indication from the representative images shown suggests that donors attach—most likely by a random, stochastic event—to the resident microcolony, are induced by proximity to the recipients, and transfer the pCF10 plasmid. The plasmid then can spread within the microcolony within the constraints of pheromone availability, metabolic and spatial factors, and positive selection pressure (Fig. 5A, B). We suggest that a ratio of ~90% recipients to 10% donors (invading donors plus transconjugants; Fig. 1A) allows for a continued basal level of induction that may benefit the entire population while limiting the costs to donors of unregulated gene expression. In the donor-dominated scenario (Fig. 5C), we suggest that recipient attachment is also a random event and that direct contact of adherent donors with invading recipient cells can lead to the induction of specific donor cells and subsequent plasmid transfer. We would expect that the relatively high steady-state ***I*** concentration in donor-dominated microcolonies might limit the subsequent spread of the plasmid beyond the initial transfer events. Notably, however, the more drastic reduction of induction and transfer (as much as 10^6^-fold) observed in vitro in donor-dominated niches^9^ may be less extensive in vivo due to non-random distribution of ***C*** and ***I*** in the GI tract and induction via direct contact in microcolonies.

## Discussion

The cumulative data presented here suggests that three major selective factors influence the composition of enterococcal microbial communities in the GI tract following the invasion of residents of one cell type by a new cell type. The strongest determinant of the final composition is the initial resident strain, which was maintained as the majority subpopulation in all experiments. The chromosomal genotype and pCF10 plasmid carriage also had significant (and independent) fitness effects influencing the final composition of the community. Pheromone regulation of transfer functions likely evolved to optimize the efficiency of the spread of the plasmid into a new host when a plasmid-carrying donor cell encounters a large population of potential recipients, and to enable both invaders and newly generated transconjugants to persist as part of a stable mixed population. Tight control of the system prevents excessive expression of costly transfer functions in scenarios where the availability of recipients is low, and minimizes costs of plasmid maintenance and expression of transfer functions to the donor while still allowing for the significant spread of the plasmid into a new genotype. A remarkable result of our study is that unmated recipient residents are not negatively impacted by the limited invasion of the donors, and may even benefit in the long term by being members of a mixed population.

## Methods

### Bacterial strains

In vivo transfer experiments employed OG1ES^18^, or OG1Sp^19^ as wild-type recipients. *E. faecalis* OG1Sp:pCF10^1^, OG1ES: pCF10^1^, or OG1Sp:pCF10:ΔprgB^13^ served as plasmid donor strains. The strains were inoculated for overnight cultures from frozen (−80 °C) stock cultures. Fluorescent protein plasmid constructs for both the inducible superfolder GFP (originally developed for *Streptococcus pneumoniae*^20^) and the donor constitutive *E. faecalis* codon-optimized tdTomato sequence have been previously published^21^. Cell walls of recipient strains were labeled with a non-toxic, blue fluorescent d-amino acid dye (7-Hydroxycoumarin-3-carboxylic acid-amino-d-alanine; 1 mM) for 3 hours in routine growth medium prior to gavage (details below) using a previously reported protocol^22^.

### Selective plates and enumeration

Recipients were quantified on tryptic soy agar (TSA) plates with 10 μg/ml erythromycin, and donors on TSA with 630 μg/ml spectinomycin plus 10 μg/ml tetracycline. Transconjugants were selected on TSA with 10 μg/ml erythromycin plus 10 μg/ml tetracycline. Each data point shown in the graphs plotted in Figs. 1 and 2 was based on at least three separate plated and counted samples.

### Gnotobiotic mice

The animal studies reported here were reviewed and approved by the IACUC committees at both the University of Minnesota (Animal Welfare Assurance number A3456) Protocol 2111-39568B. 11/10/2021–11/10/2022.) and the Mayo Clinic (Animal welfare assurance number D16-00187 - A3291-01).

Care and handling of mice were similar to the previous studies^5^. Mice were housed in the germ-free facility at Mayo Clinic Rochester in flexible film isolators. Germ-free Swiss Webster mice used for the study were obtained by breeding in larger breeder isolators and then transferring mice to smaller experimental isolators. Mice in experimental groups were age, and sex-matched. Care for the mice was provided by laboratory personnel specifically trained in the care of gnotobiotic mice following common animal husbandry practices. For dark/light cycle we employed a 12 h light cycle (6 am to 6 pm) and an ambient temperature of 67–70° F. Humidity was not controlled. Diet consisted of autoclaved Purina® chow and autoclaved drinking water. Food, water, bacterial inocula, and fecal samples were transferred in and out of the isolators through sterile ports using specialized transport material. Materials for gavaging were sterilized and placed in the isolator prior to transferring the mice to the experimental isolator. Mice were monitored daily and identified by cage number and ear punches.

### Mouse inoculation

A total of 92 gnotobiotic Swiss Webster (20–25 g) mice (equal numbers of each sex) were used in the research reported here. For each resident/invader strain combination tested we used an input of at least eight mice (four mice/cage); each cage represented a biological replicate and each mouse within the same cage represented a technical replicate. In the legends for Figs. 1 and 2, we list the number of input animals for each experiment whose results are plotted. For resident recipient experiments, mice received oral gavage with 1.3–2.1 × 10^7^ colony forming units (cfu)/mouse on day −3 and 1.7–2.0 × 10^7^ cfu/mouse donors on day 0 for all experiments. For resident donor experiments, mice received 1.8–1.9 × 10^7^ cfu/mouse donors on day −3 and 2.3–9.2 × 10^7^ cfu/mouse recipient on day 0.

### Sampling

For experiments using *E. faecalis* wild-type donor wild-type strain OG1Sp:pCF10, 12 mice were used for each of two replicate experiments. Donors, recipients, and transconjugants were enumerated from fecal samples on days 1, 4, 7, 11, 14, 18, 21, 25, 28, 32, and 35 and from GI tissue (an upper, a middle, and a lower section) on day 7 (*n* = 4) and on day 35 (*n* = 8) by selective plate quantitative culture. Note that the day 14, 21, 28, and 32 fecal collection was done only in the donor resident experiment (not in the recipient resident experiment).

For the experiments using *E. faecalis* plasmid donor OG1Sp:pCF10:ΔprgB as the invader (Fig. 1C), 10 mice were used for each of three replicates, and eight mice were used for the donor resident experiment (Fig. 1D). Donors, recipients, and transconjugants were enumerated from fecal samples on days 1, 4, 7, 11, 14, 18, and 20. Samples from GI tissue (an upper, a middle, and a lower section) were taken at 5 hours (*n* = 2), day 7 (*n* = 2), and 20 days (*n* = 4 or 6) days by selective plate quantitative culture.

Fecal samples were collected as pellet from each individual mouse at each time point and each placed in 1 ml sterile saline, homogenized, quantitatively cultured, and reported as log_10_ cfu/ml. The GI tissue sections were aseptically dissected, fecal contents removed, sliced open longitudinally, weighed, and placed into 1 ml sterile saline. They were vortexed for 30 seconds, sonicated for 5 minutes (frequency, 40 ± 2 kHz; and power density, 0.22 ± 0.04 W/cm^2^, Zenith Ultrasonic Inc., Norwood, NJ) and vortexed an additional 30 s. The sonicate fluid was quantitatively cultured and log_10_ cfu/g tissue calculated. The frequency of plasmid transfer is reported as transconjugant/donor ratio (T/D) or transconjugant/recipient (T/R) if indicated.

### Imaging

GI tissue samples were harvested from germ-free mice largely as previously published^16^. In brief, samples were aseptically grossly dissected and rapidly chilled in 2% EM-grade paraformaldehyde/KPBS. Tissue was initially fixed for ~3 h at 4 °C to improve handling and stability without excessive fluorescent protein signal degradation. After microdissection, samples were fixed in 10% normal buffered formalin for ~6 h at 4 °C, rinsed 3× in KPBS, and mounted in Prolong Glass (ThermoFisher) with a custom spacer to avoid tissue compression (Grace BioLabs). Slides were imaged on a Zeiss Axio Observer Z1 in standard confocal mode using either a ×20 (N.A. 0.8) or ×60 (N.A. 1.4) objectives under Zeiss Zen (v2.3). Post-processing was done with Huygens Professional (v 20.04; SVI) and the FIJI package (ImageJ; v1.52)^23^. Presented micrographs are representative of the full data set, and all processing was done in accordance with widely recognized microscopic data management standards^24^.

### Statistical analysis

Statistical analysis of primary enumeration data (Figs. 1 and 2) was performed using the statistics package contained in the Prism GraphPad package (v 8.0) and R (v 3.5.3). Linear regression models were fit to assess differences in slopes in the corresponding figures by adjusting for curve, day, and their interaction. In each case, the effect of the interaction term was assessed. The lower limit of detection threshold, 10 CFU, was inserted in all cases where the values were below this limit. All outcomes were log base 10 transformed to match the scaling of the corresponding figures; *p* values < 0.05 were deemed significant. Additional analyses used in the comparison of population differences at specific timepoints between experiments are described in Table 1 and Fig. 3; detailed data and analysis are in the Source Data file, and posted online as noted above.

We examined several different ways to display the enumeration data. Because the strain populations from different replicate mice and replicate cages were very similar for a given experimental condition, we chose to present the cumulative data for all mice as scatter plots to illustrate the population dynamics for all animals in each type of competition; direct comparison of results between competitions with statistical validation are presented in Table 1 and Fig. 3 as described in the text.

### Reporting summary

Further information on research design is available in the Nature Research Reporting Summary linked to this article.

## Supplementary information

Supplementary Information

Reporting Summary

## Data Availability

The detailed primary enumeration data and statistical analysis were used to generate Figs. 1–3, Supplementary Figs. 1–3, and Table 1 are available in the Source Data are provided for this paper, and also posted online at https://figshare.com/articles/dataset/Original_data_files_for_Hirt_et_al_2022_Nat_Comm_/19196624. 10.6084/m9.figshare.19196624. Source data are provided with this paper.

## Source data

Source Data
