# Supplementary material for: Dynamics of plasmid-mediated niche invasion, immunity to invasion, and pheromone-inducible conjugation in the murine gastrointestinal tract
Source: Nat Commun. 2022 Mar 16;13:1377. doi: 10.1038/s41467-022-29028-7 (PMC8927478; doi:10.1038/s41467-022-29028-7)
Supplement: Supplementary file 1 — Supplementary Information [file 41467_2022_29028_MOESM1_ESM.pdf]

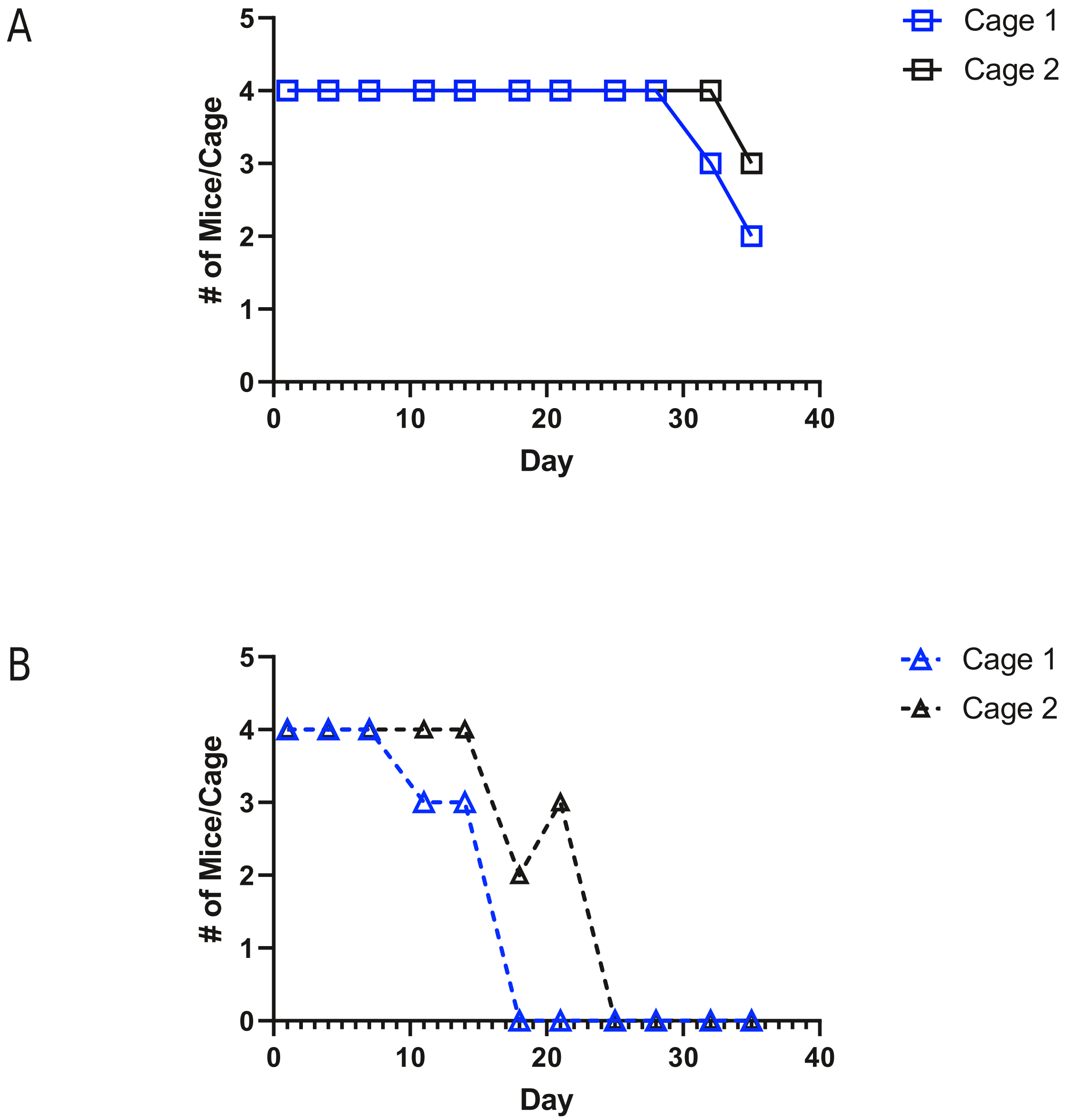

Supplementary Fig. 1. Recipients and transconjugants are eliminated in resident donor mice when OG1ES recipients invade OG1Sp:pCF10 residents (depicted in Fig. 1B of main paper).

Enumeration of recipients and transconjugants from fecal samples was carried out as described in the main paper; data from 2 cages with 4 mice/cage and limit of detection 10 CFU.

A. Number of mice with detectable recipients.
B. Number of mice with detectable transconjugants.

A

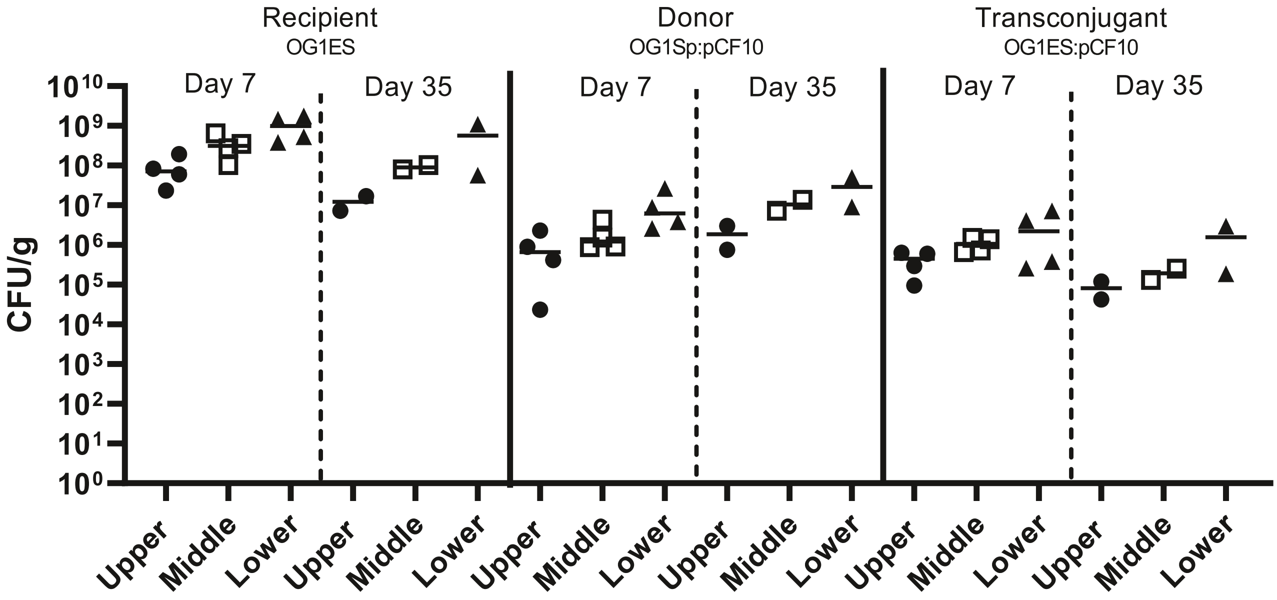

B

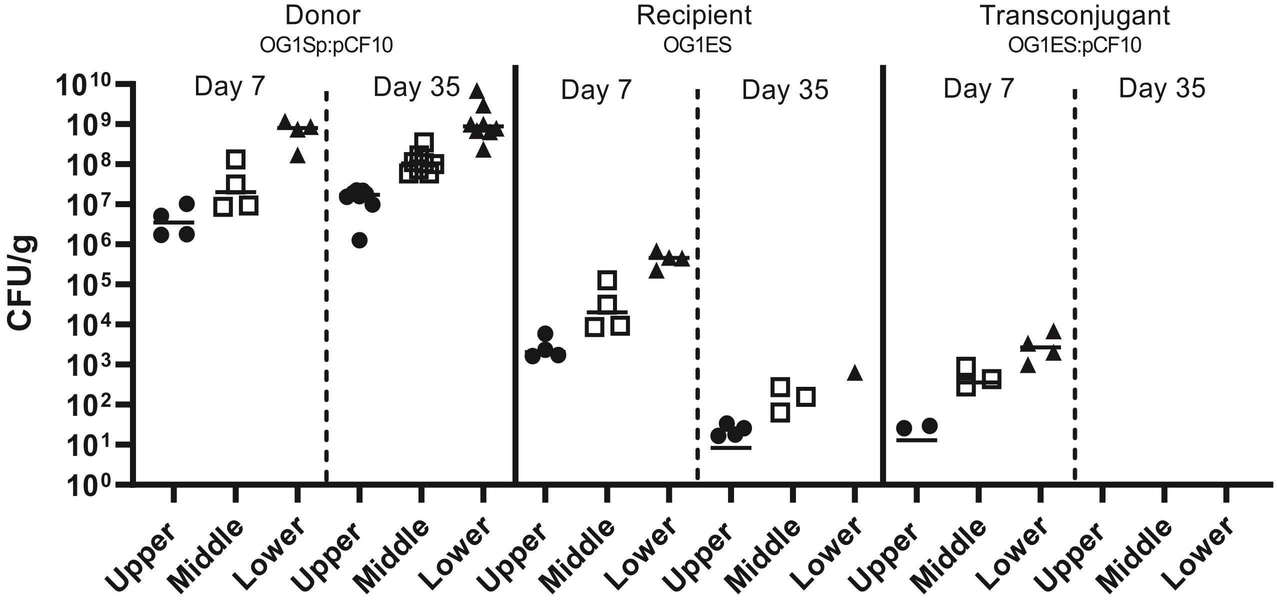

Supplementary Fig. 2. Enumeration of bacteria attached to intestinal surfaces agrees with data from fecal enumeration.

From the experiments depicted in Fig. 1A and 1B in the main paper, mice were sacrificed and bacteria attached to various segments of the intestine were enumerated by plate culture at day 7 (4 animals) or day 35 (4 animals for A, 8 animals for B). A. Resident recipient (Fig. 1A of main paper). B. Resident donor (Fig. 1B of main paper). Each point represents data from one mouse which had detectable numbers of a particular strain (transconjugants and recipients were not detectable in all mice at 35 days for B).

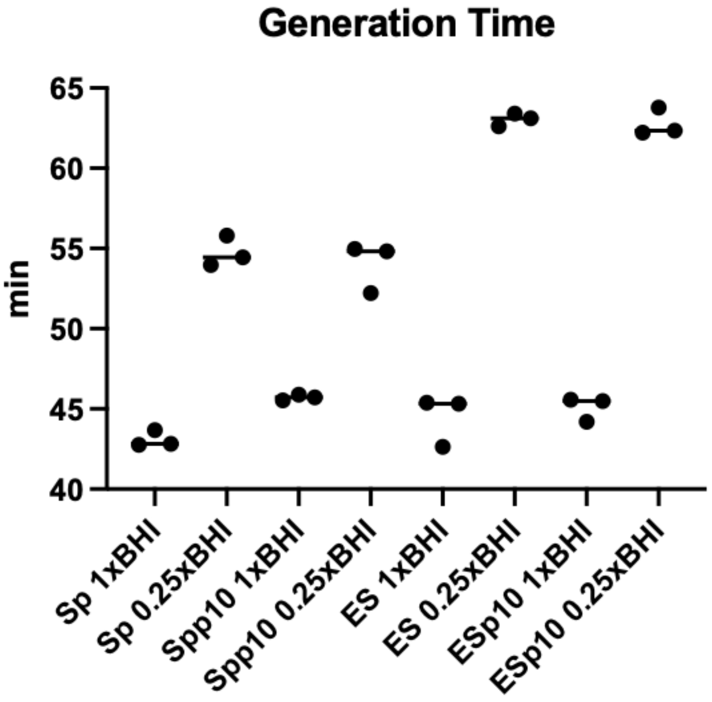

Supplementary Fig. 3. In vitro liquid culture growth of OG1SP or OG1ES strains, plasmid free or carrying pCF10 (Spp10 or ESpp10) in full strength or 0.25X strength BHI medium.

The Y-axis shows the doubling time in minutes for each strain. The results shown are based on 3 replicate growth curve experiments with each result plotted as a separate point and the mean value depicted with a line. An initial one way ANOVA (Kruskal-Wallis) gave a p-value of 0.0031. For the comparisons we used an Unpaired t-test with Welch's correction, two-tailed. These comparisons showed a significant difference in growth rates between OG1Sp and OG1ES in 0.25X strength medium ($p=0.0014$) but not in full strength medium ($p=0.2714$).

Source data are provided as a Source Data file. For full raw data, see https://figshare.com/account/projects/131918/articles/19196624 or the permanent DOI [10.6084/m9.figshare.19196624].
